# EstDZ3: A New Esterolytic Enzyme Exhibiting Remarkable Thermostability

**DOI:** 10.3389/fmicb.2016.01779

**Published:** 2016-11-16

**Authors:** Dimitra Zarafeta, Zalan Szabo, Danai Moschidi, Hien Phan, Evangelia D. Chrysina, Xu Peng, Colin J. Ingham, Fragiskos N. Kolisis, Georgios Skretas

**Affiliations:** ^1^Institute of Biology, Medicinal Chemistry and Biotechnology, National Hellenic Research FoundationAthens, Greece; ^2^Laboratory of Biotechnology, School of Chemical Engineering, National Technical University of AthensAthens, Greece; ^3^MicroDish B.V.Utrecht, Netherlands; ^4^Danish Archaea Centre, Department of Biology, Copenhagen UniversityCopenhagen, Denmark

**Keywords:** hyperthermostability, esterase, *Dictyoglomus*, functional genomics, biocatalysis, biotechnology

## Abstract

Lipolytic enzymes that retain high levels of catalytic activity when exposed to a variety of denaturing conditions are of high importance for a number of biotechnological applications. In this study, we aimed to identify new lipolytic enzymes, which are highly resistant to prolonged exposure to elevated temperatures. To achieve this, we searched for genes encoding for such proteins in the genomes of a microbial consortium residing in a hot spring located in China. After performing functional genomic screening on a bacterium of the genus *Dictyoglomus*, which was isolated from this hot spring following *in situ* enrichment, we identified a new esterolytic enzyme, termed EstDZ3. Detailed biochemical characterization of the recombinant enzyme, revealed that it constitutes a slightly alkalophilic and highly active esterase against esters of fatty acids with short to medium chain lengths. Importantly, EstDZ3 exhibits remarkable thermostability, as it retains high levels of catalytic activity after exposure to temperatures as high as 95°C for several hours. Furthermore, it exhibits very good stability against exposure to high concentrations of a variety of organic solvents. Interestingly, EstDZ3 was found to have very little similarity to previously characterized esterolytic enzymes. Computational modeling of the three-dimensional structure of this new enzyme predicted that it exhibits a typical α/β hydrolase fold that seems to include a “subdomain insertion”, which is similar to the one present in its closest homolog of known function and structure, the cinnamoyl esterase Lj0536 from *Lactobacillus johnsonii*. As it was found in the case of Lj0536, this structural feature is expected to be an important determinant of the catalytic properties of EstDZ3. The high levels of esterolytic activity of EstDZ3, combined with its remarkable thermostability and good stability against a range of organic solvents and other denaturing agents, render this new enzyme a candidate biocatalyst for high-temperature biotechnological applications.

## Introduction

Lipolytic enzymes (EC 3.1.1.x) catalyze the hydrolysis of ester bonds in lipids, and depending on their substrate preference, they are divided in two main classes, carboxylesterases (EC 3.1.1.1) and lipases (EC 3.1.1.3) (Brockerhoff, 2012). Carboxylesterases show specificity toward short to medium fatty acid chain lengths and water-soluble substrates, whereas lipases toward long-chained and water-insoluble ones (Bornscheuer, 2002; Brockerhoff, 2012). In non-aqueous media, many of these enzymes are capable of performing the inverse reaction and catalyze the synthesis of ester bonds (Bornscheuer and Kazlauskas, 2006). These characteristics, complemented by their ability to modify a very broad range of substrates with high chemo-, regio-, and enantio-selectivity, render lipolytic enzymes a very attractive class of catalysts for conducting biotransformations (Bornscheuer, 2002). Industrial applications of esterases and lipases are diverse and include the preparation of chiral compounds, the de-inking of paper pulps, the degradation of plastics, the synthesis of fine chemicals and flavoring agents, etc. (Zamost et al., 1991; Vieille and Zeikus, 2001; Kirk et al., 2002). Probably the most characteristic example of an industrially relevant esterolytic enzyme is that of the naproxen carboxylesterase from *Bacillus subtilis*, which is utilized for the biocatalytic synthesis of the non-steroidal drug naproxen (Quax and Broekhuizen, 1994).

In industrial settings, esterases and lipases are often required to perform well under harsh conditions. These include high temperatures, significant concentrations of organic solvents, metal ions, surfactants, and other agents known to cause protein denaturation and enzyme inactivation (Hough and Danson, 1999). Consequently, stability against elevated temperatures and tolerance to protein-destabilizing conditions in general, is a crucial prerequisite before the broad industrial use of this type of enzymes can be realized. During the last two decades, a growing number of thermostable enzymes that catalyze ester bond hydrolysis at elevated temperatures have been reported, mainly due to the employment of metagenomic analyses. However, hyperthermostable enzymes, i.e., enzymes that exhibit high levels of catalytic activity at temperatures above 80°C, are rarer and not many examples of such biocatalysts have been discovered and characterized.

In order to obtain new hyperthermostable enzymes, there are two main strategies, which are typically employed. The first one is protein engineering, either through rational design or directed evolution (Bornscheuer and Pohl, 2001; Dalby, 2011; Bornscheuer et al., 2012). In this approach, a mesophilic protein is optimized for stability against thermal denaturation through protein modeling-guided amino acid substitutions (rational design) or via random mutagenesis and genetic screening (directed evolution) (Matthews et al., 1987; Bornscheuer and Pohl, 2001; Lutz, 2010). The second strategy relies on the identification of naturally occurring enzymes, which have evolved to withstand high-temperature exposure by retaining their proper folding and catalytic activity under such conditions (Lorenz et al., 2002). In nature, hyperthermostable enzymes are often encoded in the genomes of hyperthermophilic microorganisms that grow optimally at temperatures above 80°C. Such organisms are encountered in all types of terrestrial and marine hot environments, and are represented only by bacterial and archaeal species. Hyperthermostable enzymes encoded in such genomes can be identified by screening genomic or metagenomic material, either bioinformatically (Levisson et al., 2007; Graham et al., 2011; Zarafeta et al., 2016) or through functional genomic screening (Ewis et al., 2004; Tirawongsaroj et al., 2008; Peng et al., 2011).

In this study, we have identified a new esterolytic enzyme, termed EstDZ3. Detailed biochemical characterization of the recombinant protein revealed that it constitutes a highly active esterase with preference toward short to medium acyl chain length substrates. The most outstanding feature of EstDZ3 is its remarkable thermostability, as it was found to retain high levels of catalytic activity even after exposure to near boiling temperatures for several hours. Importantly, this enzyme is also highly stable when exposed to high concentrations of organic solvents for extended periods of time. EstDZ3 originates from a bacterium of the genus *Dictyoglomus* and its amino acid sequence exhibits very low homology to functionally characterized proteins. Structural modeling of the new enzyme predicted that it exhibits a typical α/β hydrolase fold, which seems to include a “subdomain insertion” similar to the one present in its closest homolog of known structure, the cinnamoyl esterase Lj0536 from *Lactobacillus johnsonii*. As it was found in the case of Lj0536, this “subdomain insertion” is expected to be an important determinant of the catalytic properties of this new enzyme. The high levels of esterolytic activity of EstDZ3, combined with its remarkable thermostability and good stability against a range of organic solvents and other denaturing agents, render this new enzyme a candidate biocatalyst for high-temperature biotechnological applications.

### Environmental Sampling, Clone Isolation, and Expression Library Construction

In a previous attempt to isolate biomass-degrading thermophilic organisms, an *in situ* enrichment culture containing xanthan gum was established in a hot spring located at the Eryuan region of Yunnan, China (Menzel et al., 2015). The temperature of the sampling site when the sample was collected was 83°C and the pH about 7. After 10 days of incubation in the hot spring, a sample was collected and sealed immediately. This sample was then diluted and cultivated anaerobically at 78 and 83°C in the laboratory, as described in the “Materials and Methods” section. After three sequential passages in the same medium, the culture appeared homogeneous in morphology, with only rod-shaped cells of similar dimension visible under the microscope. Finally, the culture was diluted serially until single colonies were obtained from anaerobic Gelrite^TM^ bottles.

A single colony, termed Ch5.6.S, was subsequently isolated and cultivated under anaerobic conditions in glucose-containing medium to avoid interference of xanthan gum with DNA extraction. Sequencing of the gene encoding for the 16S rRNA revealed a 98% nucleotide identity with that of *Dictyoglomus thermophilum*, thus indicating that the isolated clone belongs to the *Dictyoglomus* genus. Then, genomic DNA derived from Ch5.6.S was isolated, partially digested, and fragments with sizes larger than 2 kb were cloned into the vector pUC18 to form a genomic library. The diversity of the generated library was ∼300,000 independent clones as estimated by the number of colonies that appeared after plating serial dilutions of the transformed *Escherichia coli* cells.

### Library Screening and Discovery of EstDZ3

**FIGURE 1:**
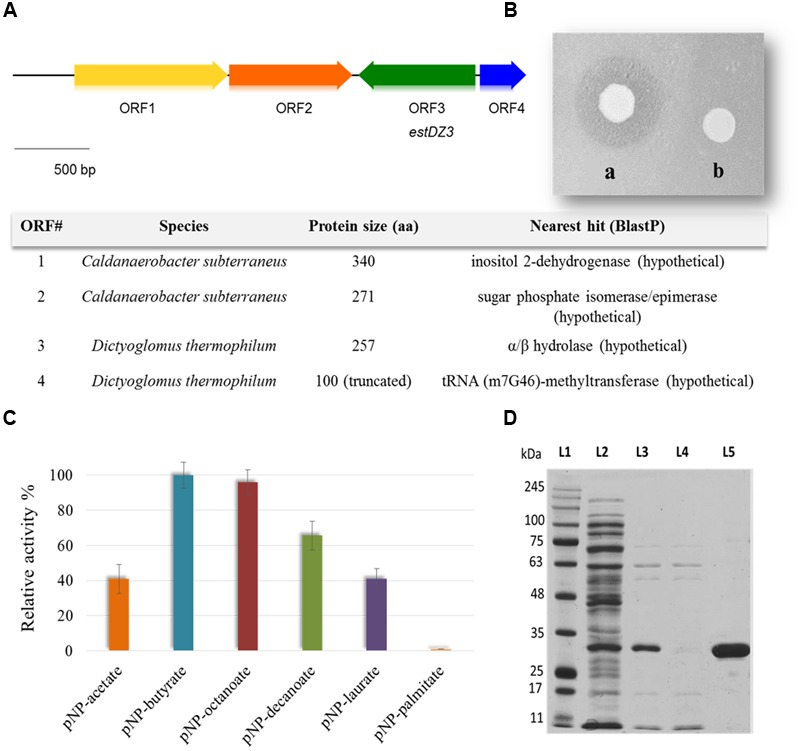
**Discovery, identification and purification of EstDZ3. (A)** Map of the 3.3 kb *ch2* insert. ORFs indicated by arrows were annotated using BlastP and the closest homolog of each ORF is listed in the corresponding table (bottom). **(B)** *E. coli* BL21(DE3) cells carrying plasmid pLATE52-EstDZ3 (a) or empty vector (b), and grown on tributyrin-enriched agar containing 0.1 mM IPTG, the inducer of *estDZ3* overexpression. **(C)** Initial substrate profiling of the esterolytic activity of EstDZ3 using clarified lysates of BL21(DE3) cells overexpressing *estDZ3.* Cell lysates were assayed against pNP esters of fatty acids with acyl chain lengths varying from C2 to C16. The relative enzymic activity was measured spectrophotometrically at 410 nm. The reported values correspond to the mean value from three independent experiments performed in triplicate and the error bars to one standard deviation from the mean value. **(D)** SDS-PAGE analysis of EstDZ3, after heat-treatment and IMAC purification. Protein bands were visualized by Coomassie staining. L1: molecular weight marker; L2: soluble lysate of *E. coli* cells producing EstDZ3; L3: soluble lysate of the same cells after heat treatment; L4: flow-through after loading of the IMAC column with the lysate corresponding to L3; L5: eluted EstDZ3 after imidazole addition. The predicted molecular weight of the recombinant enzyme is 31.6 kDa.

The generated Ch5.6.S genomic library was transformed into electro-competent *E. coli* cells and was screened for sequences exhibiting lipolytic activity by plating onto LB agar medium containing 0.1% tributyrin (Lawrence et al., 1967). After 3 days of incubation at 37°C, a zone of clearance was observed around two colonies, indicating tributyrin hydrolysis. The positive clones were re-streaked on fresh LB-tributyrin agar plates and lipolytic activity was confirmed for one of them, termed Ch2.1. The plasmid isolated from Ch2.1 was purified and the contained insert, termed *ch2*, was sequenced and found to correspond to a 3.3-kb DNA fragment, comprising four open reading frames (ORFs) that coded for the following putative proteins: (i) a hypothetical inositol 2-dehydrogenase from *Caldanaerobacter subterraneus* (ORF1), (ii) a hypothetical sugar phosphate isomerase/epimerase from *C. subterraneus* (ORF2), (iii) a predicted α/β hydrolase from *D. thermophilum* (ORF3), and (iv) a partial predicted tRNA (m7G46)-methyltransferase from *D. thermophilum* (ORF4) (**Figure 1A**).

Since the predicted α/β hydrolase was present in the selected clone as a full-length ORF and was also likely to confer the observed lipolytic activity, the corresponding gene, termed *estDZ3*, was cloned into the expression vector pLATE52 to form plasmid pLATE52-EstDZ3, which was used for heterologous expression of *estDZ3* in *E. coli*. A zone of clearance was observed around bacterial cells carrying pLATE52-EstDZ3 when grown onto tributyrin-enriched agar, in contrast to the same cells carrying an empty vector (**Figure 1B**), thus demonstrating that *estDZ3* is the gene responsible for the phenotype observed in the initial screen and suggests that *estDZ3* encodes for a protein with hydrolytic activity against tributyrin. Furthermore, when the same cell lysates were assayed for their ability to hydrolyze *p*-nitrophenyl butyrate colorimetrically, the characteristic yellow color of *p*-nitrophenol (pNP), which is indicative of ester bond cleavage, was observed only when *estDZ3* was expressed (**Figure 1C**), thus confirming that EstDZ3 is an esterolytic enzyme.

An initial substrate preference test, using soluble lysates from *estDZ3*-expressing cells and pNP esters derived from fatty acids with a range of carbon chain lengths, demonstrated that EstDZ3 has a preference for short to medium size aliphatic chains (C2–C12), while its activity is barely detectable for C16 (**Figure 1C**). This suggests that EstDZ3 acts as a carboxylesterase rather than a lipase.

### Biochemical Characterization of EstDZ3

In order to study the biochemical properties of EstDZ3, the enzyme was produced heterologously in *E. coli* and purified in soluble form. *E. coli* BL21(DE3) cells transformed with pLATE52-EstDZ3 were grown in liquid LB cultures and the production of EstDZ3 was induced by the addition of isopropyl-β-D-thiogalactoside (IPTG) as described in the “Materials and Methods” section. The recombinant protein accumulated primarily in the soluble fraction of the bacterial lysate and was purified by an initial heat-treatment step, followed by immobilized metal affinity chromatography (IMAC) to near homogeneity as evaluated by sodium dodecyl sulfate polyacrylamide gel electrophoresis (SDS-PAGE) (**Figure 1D**).

**FIGURE 2:**
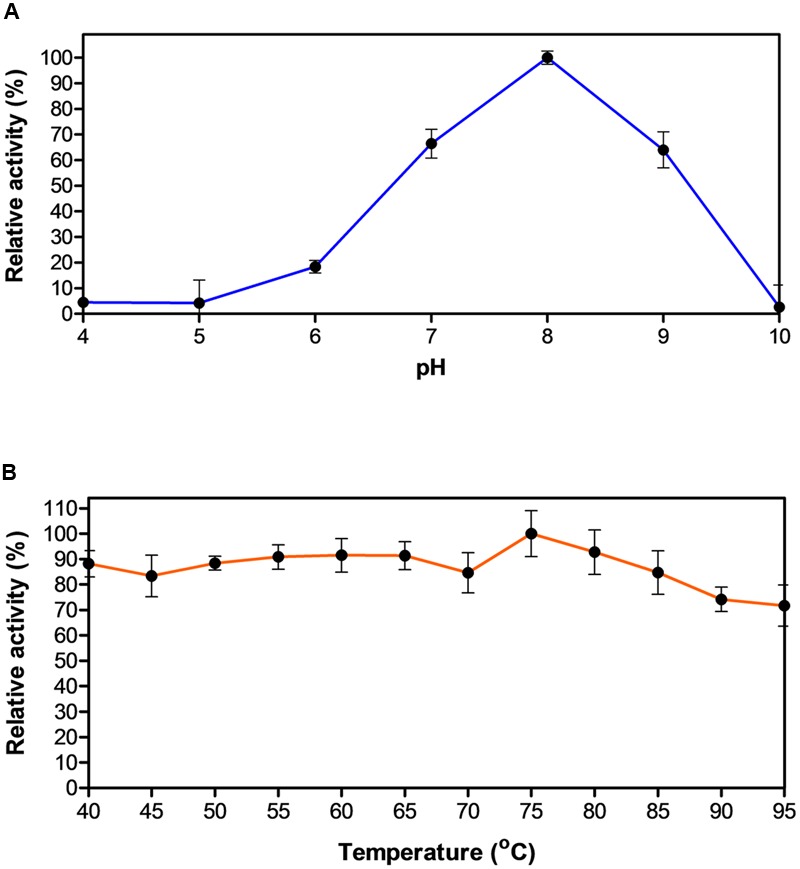
**Effect of pH and temperature on the activity of EstDZ3. (A)** Effect of pH on the activity of EstDZ3. Enzymic activity was measured in the standard reaction at 40°C for 5 min at pH values ranging from 4 to 10. **(B)** Effect of temperature on the activity of EstDZ3. Enzymic activity was measured at temperatures ranging from 40 to 95°C and pH 8. The reported values correspond to the mean value from three independent experiments performed in triplicate and the error bars to one standard deviation from the mean value.

Biochemical characterization of EstDZ3 was carried out as described in the “Materials and Methods” section using pNP-butyrate as a substrate. First, we determined the optimal pH for EstDZ3 esterolytic activity, which was assayed within the value range of 4–10 at 40°C. Significant levels of catalytic activity were recorded at pH values 7–9, with an optimum at pH 8 (**Figure 2A**). Below pH 7 and above pH 9, the esterolytic activity of EstDZ3 was rapidly diminished. Measurements of its relative catalytic activity at different temperatures, on the other hand, revealed that EstDZ3 has a very broad temperature range of action, as its esterolytic activity remained practically unchanged at temperatures between 40 and 95°C (**Figure 2B**). This type of “flat” temperature profile is quite rare but has been observed previously for esterolytic and other hydrolytic enzymes as well (Aygan et al., 2008; Novototskaya-Vlasova et al., 2012). Thus, EstDZ3 is a slightly alkalophilic and highly thermotolerant esterase.

To study the substrate specificity of EstDZ3 in more detail, we determined the catalytic parameters of EstDZ3 using a range of esters of fatty acids with carbon chain lengths, varying from C2 to C12, with pNP. EstDZ3-mediated hydrolysis of these substrates followed Michaelis–Menten kinetics and revealed that the new enzyme shows preference toward short to medium chain-length substrates (C2 and C4) (**Table 1**). The highest catalytic efficiency was detected for pNP-butyrate (C4) with a *k*_cat_/*K*_m_ value of 12,464 s^-1^⋅mM^-1^ and decreased with increasing substrate chain length for C8 and C10, while the enzyme was found to be inactive against substrates with chains longer than C12 (**Table 1**; **Figure 1C**). EstDZ3 was also found capable of hydrolyzing efficiently the longer-chain substrate pNP-laurate (C12) (**Table 1**). However, we believe that this is probably an artifact of the presence of a poly-histidine tag in the recombinant enzyme, which may be causing the specificity of the enzyme to shift toward more hydrophobic substrates as observed in a number of previous studies (Lee et al., 1999; Peng et al., 2011). Collectively, these results demonstrate that EstDZ3 acts as an esterase rather than a lipase.

**Table 1:** Kinetic parameters of EstDZ3-mediated hydrolysis against various pNP-esters.

Substrate (pNP ester)	*K*_m_ (mM)	*V*_max_ (μmol⋅min^-1^⋅mg^-1^)	*k*_cat_ (s^-1^)	*k*_cat_/*K*_m_ (s^-1^⋅mM^-1^)
Acetate (C2)	0.30 ± 0.07	355.3 ± 32.9	740	2,428
Butyrate (C4)	0.15 ± 0.02	906.4 ± 46.0	1,888	12,464
Caprylate (C8)	0.19 ± 0.01	500.6 ± 11.6	104	557
Caprate (C10)	0.17 ± 0.01	386.7 ± 8.2	80	471
Laurate (C12)	0.61 ± 0.14	357.1 ± 45.1	743	1,268

### Performance of EstDZ3 When Exposed to High Temperatures, High Concentrations of Organic Solvents and Other Denaturing Agents

**FIGURE 3:**
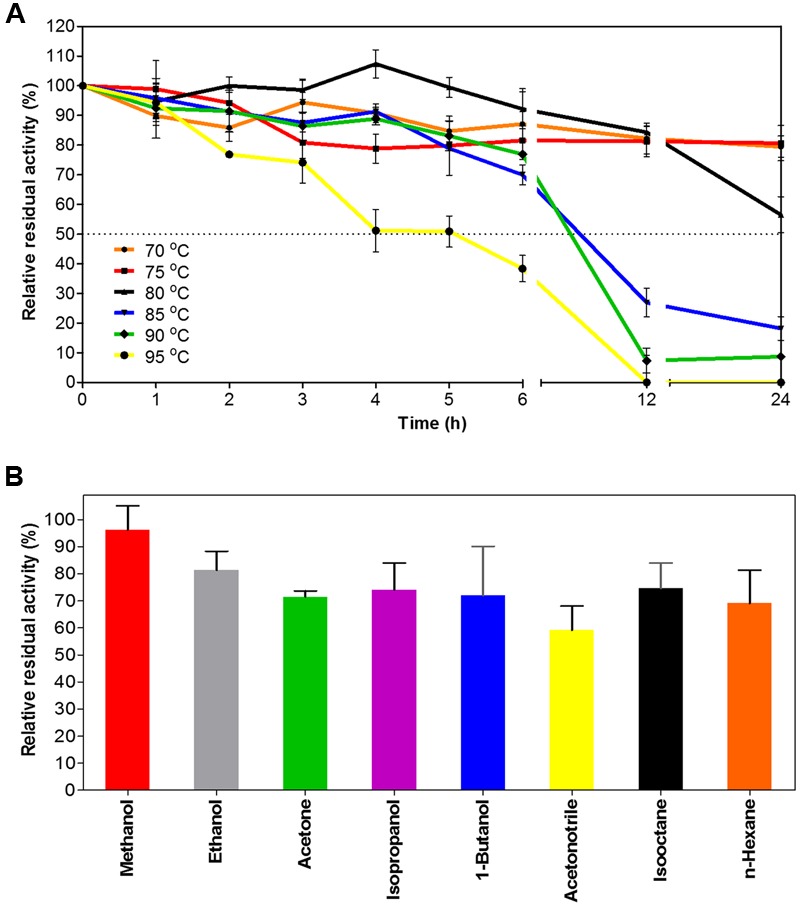
**EstDZ3 thermostability and stability against organic solvents. (A)** EstDZ3 thermostability was evaluated by measurements of residual esterolytic activity after high-temperature exposure at 70, 75, 80, 85, 90, and 95°C for up to 24 h. **(B)** EstDZ3 stability against various organic solvents was evaluated by measuring its residual catalytic activity after exposure to 50% (v/v) of the corresponding solvent for 12 h. In both panels, the reported values correspond to the mean value from three independent experiments performed in triplicate and the error bars to one standard deviation from the mean value.

When exposed to high temperatures for prolonged periods of time, EstDZ3 retained very high stability, as determined by measurements of residual levels of its catalytic activity. At 70 and 75°C, EstDZ3 esterolytic activity was practically unchanged even after 24 h of incubation, while when incubated at 80°C, the enzyme exhibited a half-life of more than 24 h (**Figure 3A**). Importantly, EstDZ3 exhibited significant levels of esterolytic activity for several hours even after incubation at temperatures as high as 95°C (**Figure 3A**). Furthermore, EstDZ3 exhibited exquisite stability against high concentrations of a variety of organic solvents. More specifically, EstDZ3 activity was found to be practically unaffected after the enzyme had been exposed to 50% (v/v) methanol for 12 h (**Figure 3B**). Similarly, when this enzyme was exposed to the same concentration of ethanol, acetone, 1-butanol, isooctane, isopropanol and n-hexane for the same period of time, its residual activity was decreased by less than 30%. Finally, after exposure to 50% acetonitrile, EstDZ3 was found capable of retaining about 60% of its maximal activity (**Figure 3B**). These results demonstrate that EstDZ3 is an esterolytic enzyme with remarkable kinetic thermostability and very good stability against prolonged exposure to high concentrations of organic solvents.

Subsequently, we studied the effects of a range of metal ions, reducing agents and detergents on the catalytic efficiency of EstDZ3. The esterolytic activity of the enzyme was practically unaffected by the addition of a variety of mono- and di-valent metals such as Na^+^, K^+^, Li^2+^, Mn^+2^, and Mg^+2^ at 1 mM concentration (**Table 2**). The addition of 1 mM Ca^2+^ and Fe^2+^ resulted in a minor decrease in its catalytic activity by about 20%, while the presence of Cu^2+^ and Zn^+2^ at the same concentration resulted in significant EstDZ3 inactivation by about 60 and 50%, respectively. When the chelating agent ethylenediaminetetraacetic acid (EDTA) was added to the reaction mixture at 1 mM, no significant change in the enzyme’s activity was observed, thus indicating that the EstDZ3 fold and/or catalytic activity does not depend on a metal co-factor, as observed, for example, in certain cases of esterolytic enzymes that resemble metallo-β-lactamases (Hermoso et al., 2005; Lagartera et al., 2005). The addition of surfactants, such as Triton X-100, Tween 20 and Tween 80 at 1% caused a reduction in the enzyme’s activity by approximately 30%, while the addition of SDS at the same concentration caused almost complete inactivation. Finally, addition of the serine hydrolase inhibitor phenylmethylsulfonyl fluoride (PMSF) resulted in a dramatic decrease in EstDZ3 activity, thus indicating that a serine residue is involved in the catalytic mechanism of this new enzyme (**Table 2**). These levels of tolerance against the presence of metals and detergents are typical for thermostable enzymes (Peng et al., 2011; López et al., 2014).

**Table 2:** Effect of metal ions, surfactants, and other chemicals on the esterolytic activity of EstDZ3.

Metal ion or chemical agent	Concentration	Relative activity (%)
None	–	100.0 ± 4.1
K+	1 mM	93.1 ± 6.7
Mn^2+^	1 mM	101.4 ± 6.2
Ca^2+^	1 mM	78.4 ± 3.0
Zn^2+^	1 mM	45.7 ± 5.7
Li^2+^	1 mM	90.8 ± 3.7
Mg^2+^	1 mM	90.1 ± 4.2
Na^+^	1 mM	90.4 ± 5.5
Fe^3+^	1 mM	78.3 ± 3.5
Cu^2+^	1 mM	40.6 ± 3.4
EDTA	1 mM	96.7 ± 5.1
PMSF	1 mM	33.8 ± 5.8
Triton X-100	1% (v/v)	67.4 ± 1.5
Tween 20	1% (v/v)	70.5 ± 5.4
Tween 80	1% (v/v)	73.2 ± 4.7
SDS	1% (w/v)	21.4 ± 2.5

Finally, EstDZ3 was found to have good tolerance against a variety of organic solvents. In the presence of 10% ethanol, acetone, and acetonitrile, the activity of EstDZ3 was slightly stimulated, whereas methanol addition at 10% had a minor inhibitory effect (**Table 3**). When either butanol, hexane or isooctane were added at 10%, EstDZ3 retained about half of its maximal catalytic activity, while isopropanol addition at the same concentration caused complete inactivation. When the concentration of methanol, ethanol, acetone, acetonitrile, isooctane, and hexane was raised to 30%, the enzyme exhibited low, but detectable levels of activity, whereas the addition of 1-butanol at the same concentration resulted in almost complete inactivation of the enzyme’s esterolytic activity (**Table 3**).

**Table 3:** Effect of organic solvents on the esterolytic activity of EstDZ3.

Organic solvent	Concentration (v/v)	Relative activity (%)
Methanol	10%	93.0 ± 6.7
	30%	26.5 ± 8.3
Ethanol	10%	114.1 ± 8.7
	30%	23.8 ± 1.5
Acetone	10%	109.6 ± 3.3
	30%	28.1 ± 7.3
Isopropanol	10%	2.8 ± 9.1
1-Butanol	10%	50.4 ± 7.9
	30%	8.3 ± 4.6
Acetonitrile	10%	109.6 ± 9.2
	30%	29.5 ± 5.4
Isooctane	10%	53.6 ± 2.9
	30%	33.6 ± 7.1
*n*-Hexane	10%	51.2 ± 2.1
	30%	25.8 ± 7.1

### Homology Analysis and Structural Modeling of EstDZ3

First, the amino acid sequence of EstDZ3 was analyzed with SignalP (Petersen et al., 2011) to detect the possible presence of protein export-signaling sequences. No such sequences were detected, thus indicating that EstDZ3 in not an exported/secreted enzyme. Then, its sequence was analyzed with BlastP against the Non-Redundant (NR) protein sequences database, the UniProtKB/SwissProt database and the Protein Data Bank (PDB). The BlastP-embedded NCBI conserved protein domain search predicted that EstDZ3 belongs to the α/β hydrolase family 5, while NR analysis revealed that EstDZ3 is identical to a putative *Dictyoglomus thermophilum* α/β hydrolase (Accession no. WP_012548346). Analysis against Uniprot/SwissProt indicated that the closest sequence homolog of EstDZ3, which has been characterized functionally, is an arylesterase from *Pseudomonas fluorescens* (sequence identity 23%, coverage 78%, Accession no. P22862.4) (Choi et al., 1990). The structure of this arylesterase has also been determined via X-ray crystallography (PDB code: 1VA4) (Cheeseman et al., 2004). The other hits from Uniprot/SwissProt included the putative peptidase YtmA from *Bacillus subtilis* subsp. *subtilis* str. 168 (Lapidus et al., 1997) (27% identity, 88% coverage), a dihydropseudooxynicotine hydrolase from *Paenarthrobacter nicotinovorans* (Baitsch et al., 2001) (23% identity, 47% coverage) and other proteins, which were either uncharacterized or with very low query coverage (<17%). On the other hand, a BlastP search against PDB yielded that the closest sequence homolog of EstDZ3, which has been characterized both biochemically and structurally is the cinnamoyl esterase Lj0536 originating from *Lactobacillus johnsonii* (identity 29%, coverage 96%, PDB code: 3PF8) (Lai et al., 2011). The rest of the PDB hits included esterases and peptidases of bacterial and archaeal origin, such as the aforementioned *Pseudomonas fluorescens* aryl esterase and a *Pyrococcus horikoshii* acylaminoacyl peptidase (PDB code: 4HXE) (Menyhárd et al., 2013).

**FIGURE 4:**
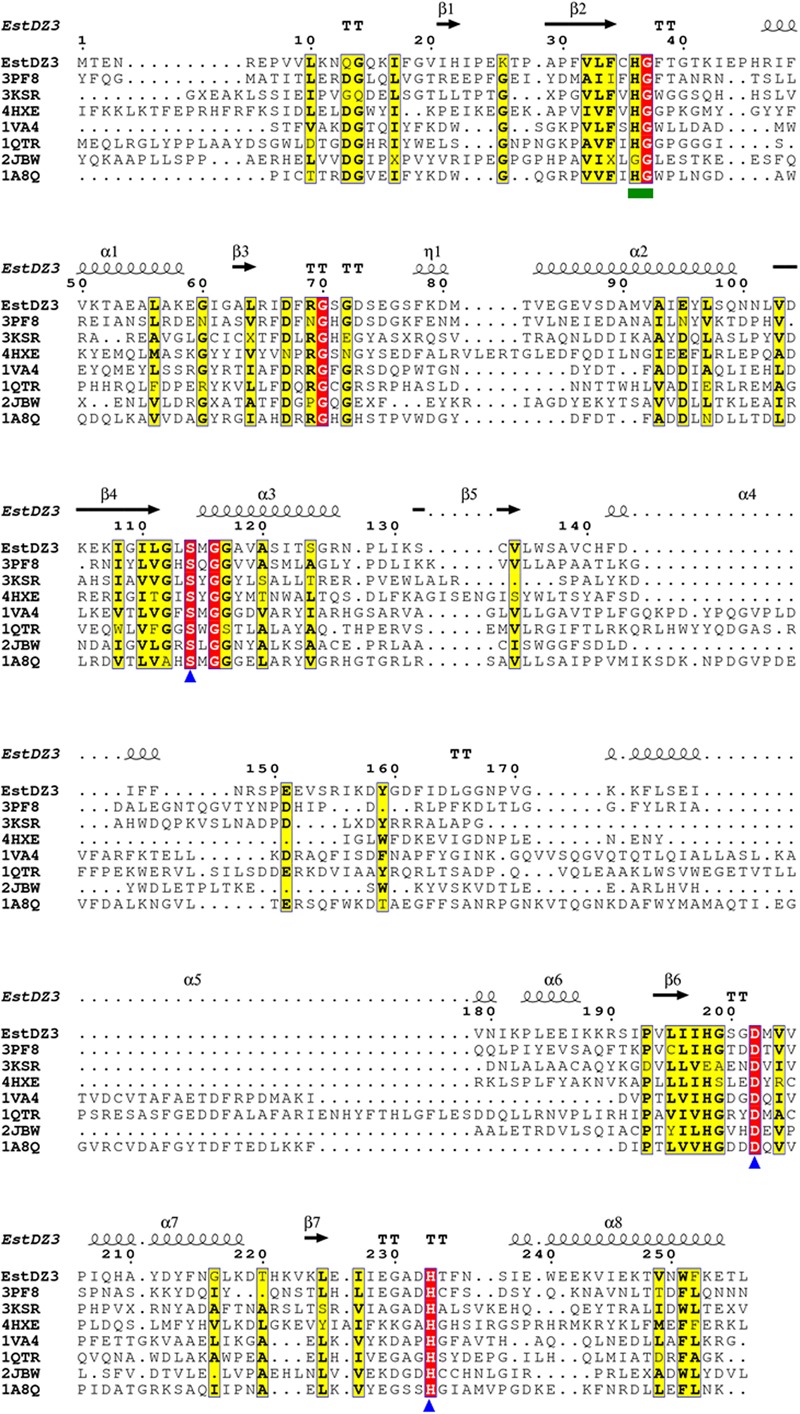
**Multiple sequence alignment of EstDZ3 and homologs with known three-dimensional (3D) structure.** The absolutely conserved amino acids are highlighted in red and similar ones in yellow. The catalytic residues, Ser114, Asp202, and His233 are indicated by blue triangles. The conserved His36-Gly37 dipeptide, which participates in the formation of the oxyanion hole during ester hydrolysis, is indicated by a green square. Elements of the predicted EstDZ3 secondary structure are denoted as α (α helix), β (β sheet), η (random coil), and T (β turn). Sequence alignment was performed using Clustal Omega (Sievers et al., 2011) and illustrated by ESPript (Robert and Gouet, 2014).

Multiple alignment of the amino acid sequence of EstDZ3 with the top seven sequences of natural proteins of known 3D structure obtained from the PDB BlastP search, indicated that EstDZ3 contains a catalytic triad comprising the residues Ser114, Asp202, and His233 (numbering for EstDZ3), which is absolutely conserved in the sequences of its homologs (**Figure 4**). Furthermore, the sequence of EstDZ3 also contains the GXSXG catalytic motif, which is very characteristic for esterolytic enzymes (Bornscheuer, 2002). Finally, the dipeptide His-Gly, which is known to contribute to the formation of the oxyanion hole during ester hydrolysis (Wei et al., 1999; Kim et al., 2013), is also present in the sequence of EstDZ3 (His36-Gly37, EstDZ3 numbering) and conserved within all of the aligned sequences (**Figure 4**).

**FIGURE 5:**
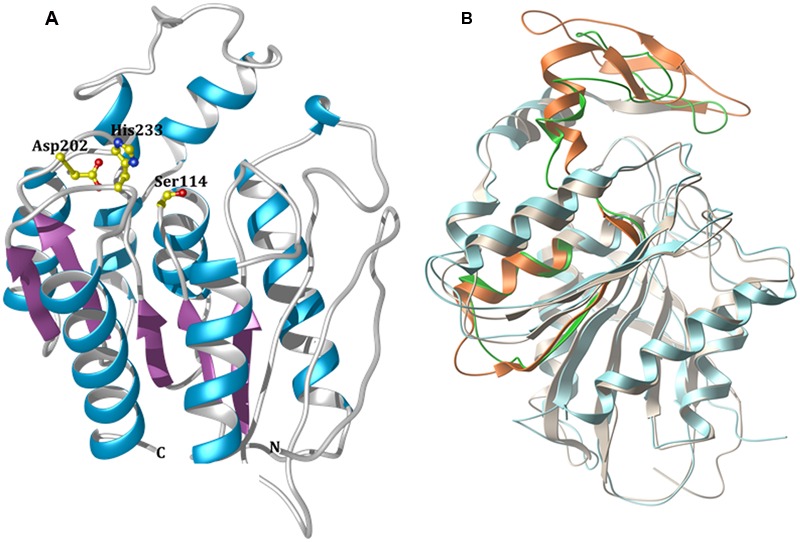
**Modeled 3D structure of EstDZ3. (A)** The modeled 3D structure of EstDZ3. Residues Ser114, Asp202, and His233, which are predicted to form the catalytic triad, are indicated in ball-and-stick representation. The figure was prepared using *Chimera* (Pettersen et al., 2004). **(B)** Superposition of the predicted EstDZ3 structure (shown in cyan) with that of its closest structural homolog, the cinnamoyl esterase Lj0536 (PDB code: 3PF8, shown in gray). The “insertion subdomain” of Lj0536 is depicted in orange, while the corresponding region in EstDZ3 is indicated in green. The figure was prepared using *MolSoft* (MolSoft LLC, 2000).

Modeling studies to predict the three-dimensional (3D) structure of EstDZ3 were performed using the I-TASSER suite (Yang et al., 2015). I-TASSER applies iterative threading assembly simulations, coupled with secondary structure enhanced Profile-Profile threading alignment and *ab initio* Monte Carlo simulations for unaligned regions. The top-ten threading templates selected by I-TASSER included esterases and peptidases, such as the *P. horikoshii* acylaminoacyl peptidase mentioned above (PDB code: 4HXE) (Menyhárd et al., 2013), the Est1E feruloyl esterase from *Butyrivibrio proteoclasticus* (PDB code: 2WTM) (Goldstone et al., 2010) and an acylaminoacyl peptidase from *Aeropyrum pernix* (PDB code: 2HU8) (Kiss et al., 2007), with sequence identities ranging from 16 to 24% and alignment coverage ranging from 84 to 96%. The presence of acylaminoacyl peptidases among the resulting threading templates is not surprising, since this type of enzymes share common sequence, structural, and functional characteristics with esterolytic enzymes. More specifically, acylaminoacyl peptidases resemble lipolytic enzymes more than classical serine proteases in terms of sequence and structure, as they also carry the GXSXG motif and adopt an α/β hydrolase fold that includes the catalytic triad Ser-Asp-His in the same sequential order that is encountered in lipases (Polgár, 1992). Furthermore, acylaminoacyl peptidases have been reported in some cases to exhibit esterolytic activity, which may surpass their peptidolytic efficiency (Polgár, 1992; Wang et al., 2006). This catalytic promiscuity has been attributed to the fact that acylaminoacyl peptidases are evolutionarily related to microbial esterases and/or lipases (Polgár, 1992). The modeled 3D structure of EstDZ3 is presented in **Figure 5A**.

The predicted EstDZ3 structure exhibits a typical α/β hydrolase fold (**Figure 5A**), which is characteristic for the vast majority of esterolytic enzymes (Bornscheuer, 2002; Brockerhoff, 2012). This provides support for the initial prediction from the NCBI conserved protein domain search that EstDZ3 belongs to the α/β hydrolase family 5. The residues Ser114, Asp202 and His233 are predicted to be located at the catalytic site, with Ser114 at the core of the highly conserved GXSXG catalytic motif (Wei et al., 1999). This is in agreement with the sequence alignment of EstDZ3 and its homologs with known 3D structure (**Figure 4**). Participation of a serine residue in the catalytic mechanism is additionally supported by the fact that the presence of the serine hydrolase-specific inhibitor PMSF (Smith et al., 1999) resulted in a dramatic reduction of the EstDZ3 esterolytic activity (**Table 2**).

Superposition of the predicted model structure of EstDZ3 with its closest sequence homolog of known structure and function, the cinnamoyl esterase Lj0536 (PDB code: 3PF8), using their corresponding secondary structure elements, demonstrated that they share a common architecture (**Figure 5B**). Compared to enzymes of the same family, the structure of Lj0536 is characterized by the “insertion” of an α/β “subdomain,” which has been found to be important for the catalytic profile of this esterolytic enzyme (Lai et al., 2011). This “subdomain insertion” appears to be present also in the predicted structure of EstDZ3. The overall conformation of this “subdomain” appears to be highly similar in the two related enzymes, differing only in the β-sheet region of the α/β “subdomain” of Lj0536 (**Figure 5B**). In the case of Lj0536, it has been found that the conformation of the “subdomain insertion” is an important determinant of the substrate specificity of this enzyme and of its close structural homologs. More specifically, when compared to its closest structural homologs in terms of substrate specificity, Lj0536 resembled mostly the aforementioned esterase Est1E from *B. proteoclasticus* (PDB code: 2WTM), which also contained a mixed α/β “subdomain” with very similar conformation (Lai et al., 2011). On the other hand, the rest of the close structural homologs of Lj0536, which contained all-α-helical “subdomains” with conformations that deviated significantly from that of the corresponding region in Lj0536, exhibited also divergent substrate specificities (Lai et al., 2011). These results suggest strongly that the presence of the “insertion subdomain” in EstDZ3 and the conformation adopted by this region of the protein is expected to be an important determinant of the catalytic properties of this new enzyme. Our computational prediction of the conformation of the “inserted subdomain” in this particular region in the EstDZ3 structure is not of sufficient accuracy –primarily due to the low sequence homology of EstDZ3 with previously studied proteins– to allow the characterization of this domain also as a mixed α/β one as in Lj0536 and Est1E, or not. The experimental determination of the 3D structure of EstDZ3 is expected to provide definitive answers to these questions.

Rationalizing the remarkable thermostability of EstDZ3 is difficult at this point. There are a number of sequence characteristics, which have been found to contribute to increased enzyme resistance against heat-induced destabilization. These include the presence of Tyr and Arg residues at higher frequencies and the presence of Ser and Cys residues at lower ones in thermophilic enzymes compared to their mesophilic counterparts (Kumar et al., 2000; Vieille and Zeikus, 2001). The sequence of EstDZ3, however, is comprised of only 1.4% Tyr and 3.2% Arg, frequencies which are lower than the average number for these amino acids in mesophilic proteins (Kumar et al., 2000). From a structural point of view, the presence of hydrogen bonds and salt bridges in surface-exposed residues and the formation of disulfides are additional factors, which have been shown to be very important contributors to enhanced thermostability in a number of cases (Kumar et al., 2000; Trivedi et al., 2006). Again, the determination of the 3D structure of EstDZ3 via X-ray crystallography, which is currently underway in our laboratories, is expected to provide explanations about the molecular determinants of the remarkable thermostability of EstDZ3.

## Discussion

The first hyperthermostable carboxylesterase was isolated from the thermoacidophilic archaeon *Sulfolobus acidocaldarius* and characterized biochemically back in 1988 (Sobek and Görisch, 1988). Since then, more hyperthermostable lipolytic enzymes have been isolated from a small number of hyperthermophiles. Quite surprisingly, very few of these are used nowadays for industrial biotransformations. Most esterases used in the industry are mesophilic, presumably due to the fact that this type of enzymes were the first to be identified and studied more extensively (Levisson et al., 2009). New enzymes with improved properties, which are continuously being discovered via metagenomic and functional genomic approaches, are being introduced into industrial biocatalytic processes only with very low frequencies. In a recent comprehensive review, Ferrer et al. (2016) have attempted to provide explanations about this apparent paradox, and have suggested three main causes: (i) the optimization phase for industrial biocatalysts is both time-consuming and expensive; (ii) the industrial criteria for the selection of appropriate biocatalysts are very strict; and (iii) patent violation restrictions are often encountered. However, as the list of new thermostable/hyperthermostable and overall tolerant lipolytic enzymes is growing, novel biocatalysts that meet the criteria for industrial use are expected to make their way into biotechnological applications. Furthermore, the discovery and characterization of a large number of enzymes with the ability to fold and retain high levels of catalytic activity under extreme conditions will broaden our understanding of their evolutionary occurrence and stabilization mechanisms and will guide future protein engineering efforts.

In this study, we have identified a new hyperthermostable esterolytic enzyme, termed EstDZ3. EstDZ3 originates from a bacterium that belongs to the *Dictyoglomus* genus and exhibits low homology to known proteins, as its closest related enzyme, which has been functionally and structurally characterized, is the cinnamoyl esterase Lj0536 from *L. johnsonii* (identity 29%, coverage 96%, PDB code: 3PF8) (Lai et al., 2011). Biochemical characterization revealed that EstDZ3 exhibits a preference toward esters of fatty acids with short to medium chain lengths, such as pNP-butyrate, indicating that it acts as a carboxylesterase rather than a lipase. Similarly to the vast majority of thermophilic esterases, EstDZ3 functions optimally at a basic pH. At its optimal conditions for ester bond hydrolysis and against its preferred model substrates, EstDZ3 presented high levels of catalytic efficiency (*k*_cat_/*K*_m_= 12,464 s^-1^⋅mM^-1^ for pNP-butyrate). Compared to the 20 esterases that have been assayed against pNP-butyrate and deposited in the BRENDA database (Schomburg et al., 2004), the catalytic efficiency of EstDZ3 is among the highest ones. More specifically, 16 out of those 20 esterases exhibited catalytic efficiencies that were one or two orders of magnitude lower than that of EstDZ3. On the contrary, comparison with the rest of the four more active esterases, EstDZ3 was found to exhibit a *k*_cat_*/K*_m_ value that is only twofold to threefold lower. EstDZ3 preference for short and medium acyl chain length substrates, such as butyric acid-based esters, complemented by its high catalytic efficiency and excellent thermostability, could be of great value for the dairy product and flavor industries (Saerens et al., 2008).

Many esterolytic enzymes lose their ability to efficiently hydrolyze esters in the presence of organic solvents, a phenomenon occurring primarily due to solvent-induced enzyme denaturation (Klibanov, 2001). On the other hand, thermostable enzymes are often capable of retaining their inherent rigidity not only when exposed to high temperatures but also against other denaturing agents, such as organic solvents (Sayer et al., 2016). EstDZ3 was found to be very stable against exposure to organic solvents, as it was capable of retaining more than 60% of its maximal activity after being exposed to high concentrations of methanol, ethanol, acetone, isopropanol, 1-butanol, acetonitrile, isooctane, and *n*-hexane for 12 h. Compared to the esterase Pf_Est from *Pyrococcus furiosus*, one of the most recently discovered hyperthermostable esterolytic enzymes (Mandelli et al., 2016), EstDZ3 exhibited higher stability when being exposed to methanol, ethanol, and isopropanol (relative residual activity 96, 81, and 74% after 12 h for EstDZ3 versus 39, 51, and 52% after 30 min for Pf_Est, respectively) (Mandelli et al., 2016), while the solvent stability of EstDZ3 resembled more those of the recently discovered organic solvent-tolerant lipase LipXO (Mo et al., 2016).

Importantly, EstDZ3 was found to exhibit remarkable thermostability, as it retained high levels of catalytic activity after exposure to temperatures as high as 95°C for several hours. Comparison with other esterases listed in a previous extensive review of enzymes derived from hyperthermophilic organisms, indicated that EstDZ3 is among the 10 most thermostable ones (Levisson et al., 2009). Only esterases of archaeal origin, such as an esterase/acylpeptide hydrolase from *Aeropyrum pernix* (Gao et al., 2003), esterases EstA and EstB from *Picrophilus torridus* (Hess et al., 2008), and four esterases from the archaeal genera *Pyrococcus* (Cornec et al., 1998; Ikeda and Clark, 1998) and *Sulfolobus* (Huddleston et al., 1995; Park et al., 2008) were reported to exhibit higher thermostability than that of EstDZ3. Among the listed bacterial esterases, EstDZ3 appears to possess the highest catalytic efficiency.

During the recent years, additional hyperthermostable esterolytic enzymes have been discovered. Some characteristic examples are a hyperthermostable lipase from *Bacillus sonorensis 4R*, which exhibits a half-life of about 2 h at 90°C (Bhosale et al., 2016), the xylan-esterase AxeA from *Thermotoga maritima* with a half-life of about 13 h at 98°C (Drzewiecki et al., 2010), and the esterase EstW from the soil bacterium *Streptomyces lividans TK64* with a half-life of 12 h at 95°C (Wang et al., 2015). The latter esterase, for which kinetic parameters have been determined, exhibits 50-fold lower catalytic efficiency compared to EstDZ3 against pNP-butyrate, and 10-fold lower against pNP-acetate, which is the preferred substrate for EstW (Wang et al., 2015).

The predicted model structure of EstDZ3 has provided preliminary insights on structural features that may be important for its function. Ongoing structural studies of the new enzyme will shed light into its physiological function and elucidate its role as a potential biocatalyst for industrial biotransformations that require high operation temperatures.

## Materials and Methods

### Reagents and Chemicals

All chemical reagents used in this study were purchased from Sigma-Aldrich. All molecular biology related products (restriction enzymes, protein markers, etc.) were from New England Biolabs unless stated otherwise.

### Environmental Sampling and Colony Isolation

A 10-ml glass bottle containing 0.1 g of xanthan gum was filled with water from a hot spring located at the Eryuan region of Yunnan, China (83°C, pH 7), sealed with an anaerobic cap carrying two needles for circulation and immersed into the hot spring. After 10 days, the bottle was collected, sealed immediately and transported to the laboratory for further cultivation. The anaerobically prepared medium contained 1 g xanthan gum (KELTROL^®^ T, food grade, Lot#2F5898K, CP Kelco), 0.13 g (NH_4_)_2_SO_4_, 0.28 g KH_2_PO_2_ and 0.25 g MgSO_4_ as well as trace elements (Na_2_MO_4_.2H_2_O, 0.025 mg; CaCl_2_.2H_2_O, 0.01 mg; FeCl_3_, 0.28 mg; CuSO_4_, 0.016 mg; MnSO_4_.H_2_O, 2.2 mg; H_3_BO_3_, 0.5 mg; ZnSO_4_.7H_2_O, 0.5 mg; CoCl_2_.6H_2_O, 0.05 mg) and vitamins (biotin 2 mg, folic acid 2 mg, pyridoxine hydrochloride 10 mg, riboflavin 5 mg, thiamine 5 mg, nicotinic acid 5 mg, cobalamin 0.1 mg, *p*-aminobenzoic acid 5 mg, lipoic acid 5 mg) per L of aqueous solution. The *in situ* enrichment was diluted 100-fold in the medium and cultivated anaerobically at 78 and 83°C. Single colonies were obtained by mixing equal volumes of serially diluted cultures and pre-warmed 1% Phytagel (Sigma-Aldrich, cat # P8170), solidification at room temperature, and incubation at 78°C. Visible single colonies were extracted from the solid medium and transferred to liquid cultures.

### Expression Library Construction

A single Ch5.6.S clone was incubated in the same medium as mentioned above, except that xanthan gum was replaced by glucose (2 g/L). Cells were harvested, DNA was extracted, and about 20 μg of genomic DNA were digested in a 400-μl reaction containing Bsp143I and Hin1II (0.02 unit/μl each) at 37°C for 30 min. The enzymes were inactivated at 70°C for 10 min and the digested DNA was precipitated and resuspended in TE buffer before gel extraction. Fragments of >2 kb were selected and mixed with pUC18 vector previously digested BamHI and SphI in a 20-μl ligation reaction (250–400 ng genomic DNA fragments, 50 ng vector, 5 units T4 DNA ligase). After overnight ligation at 14°C, 1 μl of the ligation mixture was electroporated into *E. cloni* 10G SUPREME cells (Lucigen) according to the manufacturer’s instructions. After 1 h incubation at 37°C, 10 μl of the cells were plated onto a LB agar plate containing 100 μg/ml ampicillin to estimate the size of the constructed library, and 1 ml cells were transferred to a 50 ml LB liquid culture containing ampicillin for overnight shaking at 37°C. The cells from the resulting culture were stored in 10% glycerol at -80°C.

### Screening of the Expression Library

Samples of the Ch5.6.S expression library transformed into *E. coli* strain NEB10-beta were stored in LB medium containing 20% glycerol at -80°C at a cell density of approximately 2 × 10^8^ cells/ml. For screening, this stock was diluted to 3 × 10^4^ cells/ml in LB medium and plated onto 145 mm round Petri dishes containing screening medium (LB agar containing 100 μg/ml ampicillin and 0.1% tributyrin) at a density of 10,000 colonies/plate. The plates were incubated at 37°C and the formation of zones of clearance around the colonies was monitored. Colonies that produced clear halos were purified by re-streaking on fresh screening medium. One positive clone was obtained, the corresponding plasmid was isolated, and the insert was sequenced by primer walking using the vector-specific primer M13-RP (5′-CAGGAAACAGCTATGAC-3′) and, subsequently, the insert specific primer O-034 (5′-CCGAAGAAGTGTCGAGAA-3′).

### Plasmid Construction

The coding sequence of EstDZ3 was amplified by polymerase chain reaction using the forward primer Ch2.1_f_52 (5′-GGTTGGGAATTGCAAATGACTGAAAATAGAGAACCAG-3′) and the reverse primer Ch2.1_r_11 (5′-GGAGATGGGAAGTCATTATAATGTTTCTTTAAACCAATTTACAG-3′). The PCR product was cloned into the pLATE52 vector using the aLICator ligation-independent cloning kit (Thermo Scientific) according to the manufacturer’s protocol to form plasmid pLATE52–EstDZ3.

### Protein Expression and Purification

BL21(DE3) cells carrying the pLATE52–EstDZ3 plasmid were grown in LB broth containing 100 μg/ml ampicillin at 37°C under constant shaking until the culture reached an optical density at 600 nm (OD600) of about 0.5. At that point, the expression of *estDZ3* was induced by the addition of 0.2 mM isopropyl-β-D-thiogalactoside (IPTG) followed by overnight incubation at 25°C with shaking. For EstDZ3 purification, the cells from a 500-mL culture grown in a 2-L shake flask were harvested, washed, re-suspended in 10 mL equilibration buffer NPI20 supplemented with 1% Triton X-100, and lysed by brief sonication steps on ice. The cell extract was clarified by centrifugation at 10,000 ×*g* for 15 min at 4°C and the supernatant was incubated for 30 min at 80°C in order to denature other soluble proteins. After this heat-treatment step, the precipitated material was removed by centrifugation at 10,000 ×*g* for 15 min at 4°C. The supernatant was collected and combined with 0.5 mL Ni-NTA agarose beads (Qiagen) and shaken mildly for 1 h at 4°C. The mixture was then loaded onto a 5 mL polypropylene column (Thermo Scientific), the flow-through was discarded, and the column was washed with 10 mL of NPI20 wash buffer containing 1% Triton X-100. Next, Triton X-100 was washed away by passing 10 mL of standard NPI20 wash buffer. EstDZ3 was eluted using NPI200 elution buffer. All buffers used for purification were prepared according to the manufacturer’s protocol. Imidazole was subsequently removed from this protein preparation using a Sephadex G-25 M PD10 column (GE Healthcare). Protein concentration was estimated according to the assay described by Bradford (Bradford, 1976) using bovine serum albumin as a standard. The purified protein was visualized by SDS-PAGE analysis.

### Enzyme Activity Assays

For the biochemical characterization of EtsDZ3, the catalytic activity of the enzyme was determined by quantification of the amount of pNP released from pNP-ester substrates by photometric measurement at 410 nm. The standard reaction mixture consisted of 25 mM Tris-HCl pH 8 buffer with 0.05% Triton X-100, 2 mM pNP-butyrate and 2 μg/mL enzyme and was carried out for 5 min at 75°C on a MJ Research thermal cycler, with a pre-incubation setting of the buffer to the target temperature before the enzyme was added. The reactions were terminated by placement on ice and absorbance was measured immediately using a Safire II-Basic plate reader (Tecan). Enzymic activity was recorded by measuring the absorbance of released pNP at 410 nm. All measurements were corrected for non-enzymic hydrolysis of the substrate using control reactions, where no enzyme was added and pNP standard curves were used for the calculation of the enzyme’s activity. For the temperature tolerance assay, the buffer was pre-heated and adjusted to pH 8 for each temperature tested. For the substrate specificity experiments, a range of different pNP-fatty acyl esters, such as acetate (C2), butyrate (C4), octanoate (C8), decanonate (C10) and laurate (C12) were used in concentrations ranging from 0.1 to 1 mM. For the initial substrate specificity experiments, clarified lysates of cells producing EstDZ3 were used, while blank reactions were conducted using lysate of cells carrying an empty vector. Data analysis and curve fitting to the Michaelis–Menten equation was performed using the Graphpad Prism 5 software. For the determination of the enzyme’s optimal pH, reactions were carried out at 40°C in 25 mM acetate, PIPES, Tris-HCl and glycine buffers for pH values 4–6, 7, 8–9, and 10, respectively. The extinction coefficient of pNP was determined under each reaction condition prior to the measurement. Temperature profiling of EstDZ3 was performed by incubating the standard reaction at temperatures ranging from 40 to 95°C, after the buffer was heated and titrated to the correct pH. Residual activity assays were performed by incubating the enzyme at high temperatures or 50% solvent concentration and subsequently measuring its activity into the standard reaction. Maximal (100%) enzyme activity corresponds to the activity of an enzyme sample that was not exposed to any of the tested denaturing conditions. In the case of solvent stability experiment, the incubation medium was vigorously agitated during the 12 h incubation time, and subsequently it was diluted to remove the solvent before assaying the enzyme. The assays for the determination of EstDZ3 tolerance in the presence of metal ions, detergents and organic solvents were also executed in the standard reaction with the only difference being the addition of the agents at the specified concentrations. Blanks for this experiment consisted of the same reaction mix, including the tested agent, but without the addition of enzyme. All measurements were obtained from at least three independent experiments carried out in triplicates.

### Homology Analysis and Structural Modeling Studies of EstDZ3

The EstDZ3 sequence was submitted to a similarity search analysis using BLASTp (Altschul et al., 1990) against the NR, Uniprot/SwissProt and PDB databases, and the embedded NCBI’s conserved domain search (Marchler-Bauer et al., 2014). The results obtained from the PDB search (including natural enzymes and excluding engineered ones) were aligned using Clustal Omega (Sievers et al., 2011) and illustrated with ESPript (Robert and Gouet, 2014). Modeling of the 3D structure of EstDZ3 (residues 1 to 256) was performed with I-TASSER (Yang et al., 2015). Out of the top-five predictive models prepared, the first one with *C*-score 0.3 and TM-score 0.75 ± 0.10 was selected. Superposition of the modeled structure with the closest structural homolog was performed by molecular graphics software COOT (Emsley et al., 2015) using the secondary structure elements. Molecular visualization of the modeled structure was performed with *Chimera* (Pettersen et al., 2004).

## Accession Numbers

The *estDZ3* nucleotide sequence and the *ch2* insert sequence have been deposited in GenBank under accession codes KX557297 and KX557298, respectively.

## Author Contributions

DZ, ZS, FK, and GS designed the project; DZ, ZS, HP, XP, FK, and GS designed the research; DZ, ZS, DM, HP, and EC performed the research; DZ, ZS, DM, HP, EC, XP, CI, FK, and GS analyzed the data; XP, CI, FK, and GS supervised the research; DZ, ZS, and GS wrote the paper with contributions from XP and EC. All authors read and approved the final version of the manuscript.

## Conflict of Interest Statement

The authors declare that the research was conducted in the absence of any commercial or financial relationships that could be construed as a potential conflict of interest.
